# What links BRAF to the heart function? new insights from the cardiotoxicity of BRAF inhibitors in cancer treatment

**DOI:** 10.18632/oncotarget.5853

**Published:** 2015-09-28

**Authors:** Enrico Bronte, Giuseppe Bronte, Giuseppina Novo, Fabrizio Bronte, Maria Grazia Bavetta, Giuseppe Lo Re, Giuseppe Brancatelli, Viviana Bazan, Clara Natoli, Salvatore Novo, Antonio Russo

**Affiliations:** ^1^ Department of Surgical, Oncological and Oral Sciences, Section of Medical Oncology, University of Palermo, Palermo, Italy; ^2^ Department of Internal Medicine and Cardiovascular Disease, University of Palermo, Palermo, Italy; ^3^ DiBiMIS, Section of Gastroenterology, University of Palermo, Palermo, Italy; ^4^ Department of Radiology, University of Palermo, Palermo, Italy; ^5^ Department of Medical, Oral and Biotechnological Sciences, University “G. D'Annunzio”, Chieti, Italy

**Keywords:** B-RAF, B-RAF inhibitors, cardio-oncology, cardiotoxicity, dabrafenib

## Abstract

The RAS-related signalling cascade has a fundamental role in cell. It activates differentiation and survival. It is particularly important one of its molecules, B-RAF. B-RAF has been a central point for research, especially in melanoma. Indeed, it lacked effective therapeutic weapons since the early years of its study. Molecules targeting B-RAF have been developed. Nowadays, two classes of molecules are approved by FDA. Multi-target molecules, such as Sorafenib and Regorafenib, and selective molecules, such as Vemurafenib and Dabrafenib. Many other molecules are still under investigation. Most of them are studied in phase 1 trials. Clinical studies correlate B-RAF inhibitors and QT prolongation. Though this cardiovascular side effect is not common using these drugs, it must be noticed early and recognize its signals. Indeed, Oncologists and Cardiologists should work in cooperation to prevent lethal events, such as fatal arrhythmias or sudden cardiac death. These events could originate from an uncontrolled QT prolongation.

## INTRODUCTION

Over the years the development of more and more advanced technology, applied in molecular biology, has led to the production of new molecules and an increasing use of targeted therapies. These molecules can selectively inhibit the related targets. Researchers studied extensively those targets involved in the cellular signalling pathway RAS-RAF-MEK-ERK.

RAF and its isoforms, especially the serine/threonine kinase BRAF, are commonly activated by somatic point mutations in human cancers. BRAF mutations are frequently observed in melanoma and in colorectal cancer. Activating mutations in BRAF up-regulate the downstream signalling pathway. This event stimulates neoplastic cell proliferation and decreases apoptosis [1, 2].

Throughout the years, multi-target drugs inhibiting either only BRAF or including BRAF have been developed. In this review, we discuss about those drugs suppressing BRAF, which have been approved by the FDA. They are sorafenib (2005), vemurafenib (2011), regorafenib (2012) and dabrafenib (2013). Besides, we discuss about other drugs in development, which have not yet been approved (Table 1).

**Table 1:** Overview of the BRAF inhibitors including those already approved by FDA and those yet in development

Drug	FDA approval (year)	Approved for	Phase 3 trials	Authors
Sorafenib	2005	HCC Child-Pugh A or B mRCC mDTC, refractory to radioactive iodine treatment	SHARP trial Asia-Pacific trial (NCT00492752) TARGET trial DECISION trial	Llovet JM et al.[21] Cheng AL et al.[23] Escudier B et al.[24] Brose MS et al.[28]
Vemurafenib	2011	metastatic melanoma with BRAF_V600E_ mutation	BRIM-3 trial	Chapman PB et al.[59]
Regorafenib	2012	advanced GIST previously treated metastatic colorectal cancer	GRID trial CORRECT trial	Demetri GD et al.[49] Grothey A et al.[48]
Dabrafenib	2013	metastatic melanoma with BRAF_V600E_ mutation	BREAK-3 trial	Hauschild A et al.[70]
RAF265	not approved yet (phase 1 trial)	------------	------------	------------
PLX-8394	not approved yet (phase 1 trial)	------------	------------	------------
TAK-632	not approved yet (phase 1 trial)	------------	------------	------------
MLN-2480	not approved yet (phase 1 trial)	------------	------------	------------
PLX-4720	effective in preclinical studies (but it did not reach in vivo pharmacologic levels to affect efficiently BRAF_V600E_).	------------	------------	------------
LGX818	not approved yet	------------	COLUMBUS trial (NCT01909453)	------------

## MECHANISM OF ACTION OF BRAF INHIBITORS

RAS is a proto-oncogene. Its product is a small GTPase, which interchanges the active GTP-bound state with the inactive GDP-bound state. It is the starting point of a pathway that transmits mitogenic signals from the plasma membrane to the nucleus. So, it stimulates cell differentiation and survival. The RAS family includes H-RAS, N-RAS and K-RAS proteins. Activating mutations of specific codons in these clinically relevant isoforms of RAS determine malignant transformation. They are present in a variety of human cancers. These RAS proteins are activated by growth factor receptors and stimulate their ultimate effectors through various signalling pathways. The most important pathways are the RAF/MEK/ERK pathway and the phosphatidylinositol-3-kinase (PI3K)/PTEN/Akt pathway.

The first signal transduction pathway is also called the MAPK cascade. The RAF kinases and their effectors, MEK and ERK kinases, stimulate cell proliferation or differentiation in relation to the intensity and time duration of the signal. RAF serine/threonine kinase has various isoforms, which include ARAF, BRAF, and CRAF, also called Raf-1. RAF kinases interact with the GTP-bound RAS, leading to the RAF protein kinase activation. The activated RAF phosphorylates and stimulates the kinase MEK, which in turn phosphorylates and activates the kinase ERK. This sequence of events triggers transcriptional regulators that activate a wide variety of cellular phenomena, such as cell cycle progression and cell proliferation [3-7].

The second signal transduction pathway, PI3K/PTEN/Akt, stimulates survival. PI3K is a heterodimeric protein with a regulatory subunit, p85, and a catalytic subunit, p110. When p85 binds other molecules, it is released by the inhibition of p110. PI3K localizes to the plasma membrane and it phosphorylates its substrate, phosphatidylinositol 4,5-bisphosphate (PI [4, 5] P2) on the 3′OH position to produce PI(3,4,5)P3. PI(3,4,5) P3 drags close to it, phosphoinositide-dependent kinase 1(PDK1) and Akt. PDK1 activates Akt by phosphorylating it at threonine 308. After activation, Akt leaves the cell membrane to phosphorylate intracellular substrates. Besides, it translocates to the nucleus where it stimulates several transcriptional regulators, including CREB, E2F and nuclear factor κB (NF-κB). NF-κB is localized in the cytosol constitutively linked to the inhibitory κ B protein kinase (IκB). Under activation NF-κB is translocated to the nucleus, where it stimulates the expression of several target genes, inducing cell proliferation, invasion and inflammation. Akt is involved in cell cycle progression and migration, survival, senescence, invasion, metastasis, drug resistance and DNA damage repair [8-15]. These two signal transduction pathways can interact between them and with other pathways.

BRAF mutations are frequently detectable in melanoma (50-60% of cases), papillary thyroid cancer (40-60%), colorectal cancer (about 5-10%), pilocytic astrocytoma (10-15%) and non-small-cell lung cancer (NSCLC; 3-5%). They are also present in low percentage in sarcoma, ovarian carcinoma, breast cancer and liver cancer. The six most frequent BRAF mutations are V600E, V600K, V600R, V600E2, V600D, and K601E. They are about the 95% of all BRAF mutations (COSMIC database). Among these, the most common BRAF mutation is a T1799A transversion mutation in exon 15, which has been discovered in more than 90% of BRAF-mutant tumors. This mutation determines a substitution from the amino acid valine to glutamic acid within the activation segment of the kinase. It is also called V600E. In addition, the T1799A alteration can even be associated with a second nucleotide mutation, G1798A. It leads to a V600K mutation. This mutation entails a constitutional activation of the kinase protein. So, BRAF targeting may be an incisive therapeutic tool for BRAF mutated patients. The aim of BRAF inhibitors is to suppress the hyper-activation of the signalling pathway given by the mutation of BRAF, limiting the excessive cell proliferation and balancing proliferation and apoptosis [2, 16-19].

## CARDIOTOXICITY OF BRAF INHIBITORS

### Sorafenib

To date, Sorafenib has been approved by the FDA for the treatment of patients with unresectable hepatocellular carcinoma (HCC), patients with advanced/metastatic renal cell carcinoma (RCC) and patients with locally recurrent or metastatic, progressive, differentiated thyroid carcinoma (DTC) which is refractory to radioactive iodine treatment. It is an oral drug. It inhibits multiple intracellular and cell surface kinases including VEGFR-2, VEGFR-3, RET (including RET/PTC), CRAF, BRAF and its mutant forms (including BRAF_V600E_), c-KIT, FLT-3 and platelet-derived growth factor receptor β (PDGFR-β).

There are three main phase 3 trials, which studied sorafenib in patients with advanced HCC, SHARP and Asia-Pacific trial, and unresectable and/or metastatic RCC, TARGET trial. These studies showed that among the several side effects, sorafenib gives a certain level of cardiotoxicity, mainly hypertension. In the SHARP trial, Llovet et al. studied a huge population of patients with advanced HCC. Data showed that any grade hypertension was present in 5% of patients (among the 297 patients in the sorafenib-arm), among them 2% had grade 3 hypertension, while no grade 4 hypertension was registered. Cardiac ischemia or infarction was revealed in 3% of patients. To achieve regulatory approval also in China, sorafenib had to be examined, in a parallel study, in about 200 patients with HCC from the Asia-Pacific region. This study highlighted that all grade hypertension was present in 18.8% (twenty-eight patients) and among them 2% had grade 3 or 4 hypertension. The incidence of heart attack or cardiac ischemia occurring during treatment in sorafenib-treated patients was 2.7% [9, 20-22].

Sorafenib was firstly studied in advanced RCC in the TARGET trial. 451 patients were assigned to receive continuous treatment with sorafenib and compared to the placebo-arm. In this study twenty-two patients (4.9%) were assigned to the sorafenib-arm. They reported cardiac ischemia/infarction and six events were correlated to the investigational drug. Among these patients one cardiac ischemic event led to permanent discontinuation of study drug. All grade hypertension was reported in 17% (seventy-eight patients), of which 4% had grade 3 or 4 hypertension. A larger population was then studied in North America through an expanded access program, in which about 2.504 patients were enrolled. The incidence of all-grade hypertension was 12%, and 5% was grade 3 hypertension. Schmidinger et al. in an observational, single-center study, evaluated cardiac toxicity in eighty-six patients affected by metastatic RCC. These patients were treated with either Sunitinib or Sorafenib. Among these patients only seventy-four of them were eligible for the study, 33.8% among them experienced a cardiac event (25 patients), 40.5% had ECG changes (30 patients, of which 12 experienced a cardiac event while 18 did not experience a cardiac event) and 18% were symptomatic (13 patients). However these valuations included both treatments. Giving a closer look to the Sorafenib-treated patients, it is possible to highlight that 14 patients experienced a cardiovascular event (56% upon the whole of 25 patients). Besides, among these 14 patients, six patients had ECG changes. Eight patients had symptoms likely related to myocardial damage. Furthermore, the use of Sorafenib led to cardiac ischemia in 3% of patients [9, 23-25].

The recent prospective, open-label, non-interventional, non-controlled, multicenter study conducted in 18 countries, which is called PREDICT study, enrolled 2855 patients with advanced RCC. All of them were treated with Sorafenib. It registered, among the drug-related adverse events, only hypertension, which was present in 4.2% (110 patients) [26].

It is more recent the phase 3 trial, which led to the approval of Sorafenib in locally advanced or metastatic differentiated thyroid cancer. The DECISION trial is a multicentre, randomised, double-blind, placebo-controlled study, in which patients were assigned on a 1:1 basis to Sorafenib or placebo, so 207 patients were randomised in the Sorafenib-arm. Any grade hypertension was present in 40.6% (84 patients), of which 9.7% was grade 3 (20 patients), while no grade 4 hypertension was registered. As regards dyspnoea, any grade was highlighted in 14.5% (30 patients), grade 3 in 4.8% (10 patients). No grade 4 dyspnoea was registered. Among serious adverse events occurring in 2% or more of patients receiving Sorafenib, there was dyspnoea in 3.4% (7 patients among 207). One death, in the Sorafenib group, was attributable to myocardial infarction [27].

Many other studies evaluated the safety profile of Sorafenib compared to either placebo or another drug (including sunitinib, axitinib, brivanib, tivozanib, dovitinib and linifanib). All the studies reported hypertension, all grade hypertension ranging from 17.5% to 34% (with a mean value of about 26%). More heterogeneous values were recorded as regards grade 3/4 hypertension ranging from 1% to 18% (with a mean value of about 10%). Most of these studies registered asthenia, all grade asthenia ranging from 11.4% to 17% (mean value of about 14%) and grade 3/4 asthenia ranging from 2.1% to 5% (mean value of about 3.5%). Very few cases led to discontinuation of treatment with Sorafenib. The studies also reported other cardiovascular adverse events that were present in low percentage or in individual cases, such as myocardial ischemia, cardiac failure, pulmonary embolism, peripheral edema and cerebrovascular accidents. Two studies reported dyspnoea but they registered very different percentages. One of them reported all grade dyspnoea in 9% of patients of which 2% had grade 3/4. The other one reported all grade dyspnoea in 20% of patients, of which 7% had grade 3/4 [28-36].

Similar results about hypertension were observed in a meta-analysis by Funakoshi T et al. All grade hypertension was present in 23.1% of patients. High-grade hypertension occurred in 6.0% [37].

Another meta-analysis by Choueiri et al. evaluated the incidence and the risk of arterial thromboembolic events (ATEs) linked to Sorafenib. ATEs had an incidence by 1.7%, while it was calculated an RR of ATE by 3.1 [38].

In a phase 1 open-label study Tolcher et al. analysed the cardiovascular safety of Sorafenib. They evaluated the baseline and the variations of QTc interval, together with other parameters such as left ventricular ejection fraction (LVEF), blood pressure and heart rate. Thirty-one patients, after one cycle of Sorafenib treatment, showed a modest prolongation of the QT/QTc interval. They registered mean increases from baseline by 9.0 and 4.2 milliseconds (ms) respectively for QTcF and QTcB. None of the patients had a QTcB or QTcF value >500 ms at any time of the study. Besides none of them showed a change from baseline in QTcB or QTcF ≥60 ms. Only one patient had a prolongation in either QTcB or QTcF that was a +50 ms change from QTcF baseline. Besides, this study highlights that there are few cases in which the mean maximal increases from baseline can be of 16 ms for QTcB and 20 ms for QTcF. This was showed in continuous treatment with Sorafenib. So these results reveal that there is no clinically relevant effect of Sorafenib on cardiac repolarization, if it is used at therapeutic doses (400 mg BID) [39].

Sorafenib was firstly created to inhibit RAF kinases. It strongly acts on Raf-1 and BRAF more than the other targets it has. This could be underlined taking a look to the IC_50_ it has towards its targets. IC_50_ is the half maximal inhibitory concentration at which the compound reaches half of its maximal inhibitory effect. Raf-1 is affected with a mean IC_50_ of 6 nM with a standard deviation (SD) of ± 3; BRAF wild-type: 25 ± 6; its mutant form BRAF_V600E_: 38 ± 9; VEGFR-2: 90 ± 15; murineVEGFR-2 (flk-1): 15 ± 6; mVEGFR-3: 20 ± 6; mPDGFR-β: 57 ± 20; Flt-3: 58 ± 20; c-KIT 68 ± 21. A study evaluated the interaction between several kinases and thirty-eight kinase inhibitors. In this study Sorafenib strongly bound to 38% of the tyrosine and 11% of the serine/threonine kinases. The kinase binding dissociation constant (K_d_ - which is commonly used to describe the affinity between a ligand and a protein) was less than 3 μM. This concentration is under therapeutic plasma levels. Besides, only 16% of the tyrosine kinases and 1% of the serine/threonine kinases bound Sorafenib with a K_d_ of less than 100 nM. This is the value under which there is a greater binding affinity between the drug molecule and the ligand. In fact, the lower the value of K_d_ is the higher the binding affinity will be, and vice versa. So, even though sorafenib was initially described as a potent inhibitor of RAF serine/threonine kinase, it preferably binds tyrosine kinases and in some cases with affinities within ten-fold of that for its expected primary target [40-42].

### Regorafenib

The kinase inhibitor Regorafenib has been approved for the treatment of patients affected by metastatic colorectal cancer (CRC). Patients already treated with fluoropyrimidine-, oxaliplatin- and irinotecan-based chemotherapy, an anti-VEGF therapy, and also an anti-EGFR therapy, if the patients have a wild type KRAS mutational status. It has also been approved for locally advanced, unresectable or metastatic gastrointestinal stromal tumors (GIST) in patients who have been previously treated with imatinib mesylate and sunitinib malate. Regorafenib is a small molecule. It inhibits several membrane-bound and intracellular kinases involved both in normal cellular functions and in pathologic processes such as oncogenesis, tumor angiogenesis, and maintenance of the tumor microenvironment. It suppresses the action of several targets such as RET, VEGFR-1, VEGFR-2, VEGFR-3, KIT, PDGFR-α, PDGFR-β, FGFR-1, FGFR-2, TIE2, DDR2, TrkA, Eph2A, Raf-1, BRAF and its mutant form BRAF_V600E_, SAPK2, PTK5, and Abl [43-45].

There are two main phase 3 trials that led to the approval of regorafenib by the FDA. The CORRECT trial studied patients affected by metastatic CRC. The GRID trial studied patients affected by advanced GIST. In the CORRECT trial, among the 500 patients treated by regorafenib, the most present cardiovascular side effect was hypertension. Any grade hypertension emerged in 28% of the patients, grade 3 hypertension emerged in 7% of the patients, while no grade 4 hypertension was highlighted. The other cardiovascular side effect, which came to light, was dyspnoea. Any grade dyspnoea was present in 6% of patients (28 patients) including only one patient experiencing grade 3 dyspnoea (< 1%). No grade 4 dyspnoea was reported. Among patients assigned to regorafenib-arm, 12 patients (2%) had thromboembolic events. Besides, regorafenib increased the incidence of myocardial ischemia and infarction 1.2% versus 0.4% in the placebo-arm. In the GRID trial, 132 patients were treated with regorafenib. Among them 48.5% had any grade hypertension (64 patients), 22.7% presented grade 3 hypertension (30 patients), while only one patient (0.8%) had grade 4 hypertension. So, among grade ≥3 side effects, hypertension was the most common (23.5%). This adverse event could be managed with dose modification and appropriate anti-hypertensive intervention. One patient, in the regorafenib-arm, had cardiac arrest, which was considered by the investigators a grade 5 adverse event drug-related [9, 46-48].

Regorafenib has also been studied for advanced HCC in a phase 2 trial. It reported any grade hypertension in 36% of the patients (13 patients), of which one patient (3%) had grade ≥3 hypertension. One patient (3%) had grade ≥3 arrhythmia, which led to discontinuation of treatment [49, 50].

In an open label, single arm study, 25 patients with advanced solid tumors assumed regorafenib at multiple doses. In this study the effect of multiple doses on the QTc interval was evaluated. No large changes in the mean QTc interval (> 20 msec) were detected in the study.

Regorafenib is biochemically similar to sorafenib but essentially different, by showing a more powerful inhibition on oncogenic kinases. This aspect emerges from its biochemical profile, namely the IC_50_ on each target kinase. VEGFR-1 has an IC_50_ of 13 nM with a SD of ± 0.4, murineVEGFR-2: 4.2 ±1.6, murineVEGFR-3:46 ± 10, TIE2: 311 ± 46, PDGFR-β: 22 ± 3, FGFR-1: 202 ± 18, KIT: 7 ± 2, RET: 1.5 ± 0.7, Raf-1: 2.5 ± 0.6, B-RAFwt: 28 ± 10, BRAF_V600E_: 19 ± 6. These target kinases were also analyzed in mechanistic cellular phosphorylation assays. They evaluated the inhibition of receptor auto-phosphorylation in cells, which expressed FGFR, PDGFR-β, BRAF, VEGFR-2, VEGFR-3, TIE2. Regorafenib potently inhibited VEGFR-2 and TIE2 auto-phosphorylation in cells with an IC_50_ respectively by 3 nM and 31 nM. It inhibited PDGFR-β auto-phosphorylation with an IC_50_ of 90 nM, while FGFR signalling with a value of about 200 nM. Finally, the inhibition of the MAPK signalling pathway was evaluated using tumor cells, which expressed wild-type KRAS and BRAF, and in melanoma cell line BRAF_V600E_ mutated. The first ones had an IC_50_ by 380 nM, while the second ones had an IC_50_ by 272 nM. So, the potency of inhibition from regorafenib expressed in the cellular assays was correlated to the one showed in biochemical assays, with some exceptions. TIE2 showed an inhibition of about 10-fold weaker in biochemical assays than the cellular auto-phosphorylation assay. On the contrary, BRAF and BRAF_V600E_ highlighted an inhibition of about 14-fold stronger in biochemical assays than the cellular auto-phosphorylation assay [51, 52].

### Vemurafenib

Vemurafenib is a kinase inhibitor. It has been approved for the treatment of patients with unresectable or metastatic melanoma with BRAF_V600E_ mutation. It is an orally administered drug, which potently inhibits some BRAF serine-threonine kinases with activating mutations and in particular BRAF_V600E_. Besides, vemurafenib has shown *in vitro* inhibitory activity on other kinases such as CRAF, ARAF, wild-type BRAF, SRMS, ACK1, MAP4K5 and FGR [55-57]. Vemurafenib inhibits BRAF_V600E_ with an IC_50_ of 31 nM, 48 nM for CRAF, 100 nM for wild-type BRAF. It inhibits SRMS with an IC_50_ of 18 nM, 19 nM for ACK1, 51 nM for MAP4K5 and 63 nM for FGR [55].

Three studies represent the cornerstone in the approval of vemurafenib by the FDA. These are BRIM-1 (phase 1 trial), BRIM-2 (phase 2 trial) and most importantly the BRIM-3 (phase 3 trial). In the BRIM-3 trial, vemurafenib was compared to dacarbazine in 675 patients with previously untreated metastatic melanoma BRAF_V600E_-mutated. In this study there were four deaths (1%) in patients treated by vemurafenib not directly attributed to disease progression, which occurred within 28 days from the last dose administration of the investigational drug. These deaths were linked to fatal adverse events, which were cerebrovascular accident, pneumonia, cardiopulmonary failure and aortic aneurysm rupture. But, none of them were attributed to vemurafenib. Grade 1-4 asthenia was recorded in 36 patients in vemurafenib-arm (10.7%). The QT interval was examined in a sub-study within BRIM-2, showing that for this drug there is a concentration-dependent increase in QT interval [54, 56, 57].

In the Expanded Access Program conducted in the United States patients with metastatic melanoma were treated with vemurafenib. Among these patients twenty-four (7%) had an increase in QTc interval of more than 480 milliseconds. Eleven patients (3%) had QTc intervals of more than 500 milliseconds. Nineteen patients (5%) got an increase in QTc interval from baseline by at least 60 milliseconds. But, it has to be noted that none of these QTc interval prolongations was associated with any significant clinical finding, such as arrhythmia. Two patients reported a prolonged QT interval, which was a treatment-related serious adverse event. Besides in two cases (0.5%) the prolonged QT interval led to vemurafenib permanent discontinuation [58].

Larkin et al. studied in an open-label, multicentre, safety study, 3222 patients with BRAF_V600_ mutated metastatic melanoma, who received at least one dose of vemurafenib. Among these patients 316 (overall - 10%) experienced prolonged QT interval with or without clinical manifestation, such as atrial fibrillation, sinus tachycardia, atrial flutter and other atrial arrhythmias, and ventricular arrhythmias. Grade 1 and 2 QT prolongation was present in 287 patients (9%), while 52 patients (2%) had corrected (Fridericia) QT interval (QTcF) prolongation of more than 500 ms (grade 3 and 4). Peripheral edema was present in 215 patients (7%), including 212 with grade 1 and 2, while 5 had grade 3 and 4. Hypertension was also registered, Grade 1 and 2 in 117 patients (4%), while grade 3 and 4 in 76 (2%) with an overall percentage by 6%. Four patients died because of cerebrovascular accident and other four patients died because of pulmonary embolism. The most common adverse event leading to drug discontinuation included QTc prolongation in nine patients (<1%). Among these ones only two had QTcF longer than 500 ms, dyspnoea in six (<1%) and cerebral haemorrhage in six (<1%) [59].

More recently Larkin et al. evaluated the efficacy of the combination therapy with vemurafenib and cobimetinib in comparison with vemurafenib plus placebo. Among the 239 patients treated with vemurafenib plus placebo grade 1 QT-interval prolongation was registered in 8 patients (3%), grade 2 in 2 (1%), grade 3 in 3 (1%); no grade 4 toxicity was registered. Low percentage of frequency was also reported for decreased ejection fraction, there was no grade 1 or 4 toxicity, grade 2 was present in 4 patients (2%), while grade 3 was present in 3 (1%) [60].

Besides, some medical cases report cardiovascular toxicity by vemurafenib. They report about not only QT-interval prolongation, but also about pericarditis and some of them with effusion and tamponade [61, 62].

### Dabrafenib

Dabrafenib is a potent and selective inhibitor of some mutated forms of BRAF kinases. It has been approved as a single agent or in combination with trametinib for the treatment of patients with unresectable or metastatic melanoma with BRAF_V600E_ mutation (and also metastatic melanoma with BRAF_V600K_ mutation for the combination therapy). It is not indicated for treatment of patients with wild-type BRAF melanoma. It inhibits *in vitro* and *in vivo* BRAF mutated forms with an IC_50_ by 0.65 nM for BRAF_V600E_, 0.5 nM for BRAF_V600K_, 1.84 nM for BRAF_V600D_. Less potently it inhibits CRAF with an IC_50_ of 5.0 nM and wild-type BRAF kinases with an IC_50_ of 3.2 nM and at greater concentrations other kinases such as SIK1, NEK11, and LIMK1 [63].

The clinical trials, which led to the approval of this drug by the FDA are called BREAK. They are BREAK-1 (phase 1 trial), BREAK-2 (phase 2 trial), BREAK-3 (phase 3 trial) and BREAK-MB (phase 2 trial in BRAF-mutant melanoma metastatic to the brain). No significant cardiovascular toxicity was reported in these trials, in which patients were treated with dabrafenib as a single agent [64-67].

Cardiovascular adverse events were recorded in patients which underwent the combination therapy dabrafenib plus trametinib. These adverse events were venous thromboembolism, decreased ejection fraction, pulmonary embolism, cardiomyopathy, hypertension and hypotension, which were present in low percentages. But, it is not possible to determine which one of the two drugs is more cardiotoxic than the other one or if it is the combination therapy itself that determines cardiotoxicity [68-70].

### Drugs in development

Many other molecules are still in development. Most of them are so far studied in preclinical level, while others have reached phase 1 trials. So, they still need more data in order to establish if they determine cardiotoxicity and eventually which level of expression. They include RAF265, an orally bioavailable small molecule. It inhibits the activities of several intracellular kinases such as BRAF_V600E_, wild-type BRAF, CRAF, VEGFR2, PDGFR, RET, c-KIT, SRC and other targets with an IC_50_ ranging from less than 20 to more than 100 nM. It inhibits more potently BRAF_V600E_ and VEGFR2 than the other targets. Among these molecules there are also PLX-8394, TAK-632 (which is a potent and selective pan-RAF inhibitor that overcomes paradoxical RAF activation), MLN-2480. The molecule PLX-4720 was one of the first highly selective inhibitors of mutant BRAF_V600E_ (IC_50_ = 13 nM). It was effective on cell lines in preclinical studies, but it did not reach *in vivo* pharmacologic levels to affect efficiently BRAF_V600E_ [16, 71-77].

Finally, LGX818 a highly potent BRAF inhibitor has been studied and developed for BRAF-mutated advanced melanoma. It has a much longer half-life of dissociation from BRAF_V600E_ kinase than the other molecules. It emerged from the studies that its toxicity profile was similar to those of vemurafenib and dabrafenib. At present, there is an ongoing trial. The COLUMBUS trial is a randomized, open label, 3-arm phase 3 study which compares the efficacy and safety of LGX818-MEK162 combination or LGX818 monotherapy to vemurafenib in patients with unresectable or metastatic melanoma with BRAF_V600_ mutation (NCT01909453) [78].

## HYPOTHESES FOR ANTI-BRAF-RELATED CARDIOTOXICITY

The human ether-a-go-go related-gene K^+^ channel hERG is encoded by the KCNH2 gene. It is a voltage-activated K^+^ channel responding to changes in membrane potential, widely expressed in the heart. It has a critical role in the repolarization process of the action potential in cardiomyocytes. In fact, these channels are responsible of the rapidly activating delayed rectifier K^+^ current (I_Kr_) in the heart. The outward K^+^ currents and in particular the delayed rectifier repolarizing current I_K_ correlates with repolarization. This determines the configuration of the action potential. It results from the sum of two different types of K^+^ currents: the rapid component, I_Kr_, and the slow component, I_Ks_. These currents are fundamental in the transition from phase 2 to phase 3 of the action potential in cardiomyocytes. So, small changes in conductance can significantly modify the effective refractory period, hence the action potential duration. A reduction or an increase in I_Kr_ lead respectively to long or short QT interval converting in a QT syndrome. So, both of these conditions render liable affected hearts to fatal arrhythmias (including torsades de pointes, TdP) and sudden cardiac death. The hERG channels could be also up-regulated by the signalling of growth factors and contribute to it. Among these factors there is BRAF, which may participate in the regulation of these channels. Cardiac cells express all the three RAF family members, RAF-1, BRAF, and ARAF. They stimulate survival and growth of cardiomyocytes. A recent study has shown that BRAF is a powerful regulator of hERG K^+^ channels, stimulating them. Wild-type BRAF increased hERG channel protein abundance in the cell membrane and consequently it increased the hERG-mediated current through the cell membrane. Furthermore, cells treated with the BRAF inhibitor PLX-4720 highlighted a down-regulation of hERG channel protein quantity and activity. Hence, it is possible to consider that BRAF inhibitors down-regulating hERG channels protein quantity and down-regulating their activity, undermine I_Kr_. This event determines a slowdown in repolarization, which leads lastly to QT prolongation [79-84].

**Figure 1:**
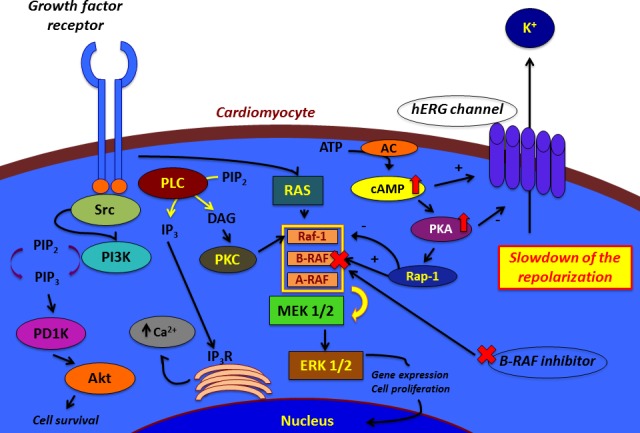
Hypotheses for the effects of BRAF inhibitors on cardiomyocyte

It remains to understand the mechanism by which BRAF acts on hERG channels. Studies revealed that in some cells cAMP stimulates MAPK activation in the presence of BRAF and Rap1. In fact, cAMP activation stimulated PKA, which in turn activated BRAF through the small G protein Rap-1. The latter is a selective activator of BRAF and inhibitor of Raf-1. Other studies disclosed that increased concentration of cAMP determines effects on hERG function through two pathways. Although it increases hERG protein abundance and the channel production, it decreases the trafficking velocity. On one hand, cAMP stimulates PKA. The latter directly phosphorylates hERG. PKA-dependent phosphorylation of hERG gives the channels a minor ability to open during action-potential voltages. So, there is an inhibition of K^+^ current directed towards the outside of the cell at all voltages. On the other hand, cAMP directly binds to the hERG protein and shifts the voltage-dependence of activation to depolarized potentials. Even though this direct effect counterweights the PKA-dependent action, the current inhibition and accelerated deactivation remain unopposed. The final effect is a reduction of hERG current. So, it could be possible to hypothesize that BRAF inhibition through an inhibitory molecule determines a compensatory up-regulation of cAMP. The increase in cAMP concentration leads to the stimulation of PKA, whose action stimulates the phosphorylation of hERG channels. This event gives the channels a minor capability to open and facilitate the repolarization of the cell. The slowdown of the repolarization implies QT prolongation [85-90] (Figure 1).

## CONCLUSIONS

The RAS/RAF/MEK/ERK pathway has a central role in many cellular processes. It transmits a mitogenic signal and stimulates cell proliferation, gene expression and survival. The molecules involved in this pathway have been investigated for many years either in their wild-type or mutated form. The evidence of BRAF mutated forms (above all BRAF_V600E_) in melanoma, papillary thyroid cancer and colorectal cancer have pushed the researchers to focus on this target. Over time various molecules have been developed, first as multi-target agents but progressively the aim has become to produce drugs that could selectively target BRAF and particularly its mutated forms. These selective molecules for BRAF mutated forms have shown to impair QT interval throughout their development. For this reason a precaution for the use of BRAF inhibitors in patients with pre-existing conditions affecting their QT interval on ECG has been inserted in their label.

In this work we analyze the cardiotoxicity data related to molecules that target either as multi-target or selective agents (Table 2). The data analysis shows that multi-target molecules have somewhat an influence on QT interval, even though clinically not significant. Vemurafenib, as a selective agent, has a more consistent influence on QT interval leading to clinically significant changes. Conversely the recently approved BRAF inhibitor dabrafenib seems to have a slight cardiotoxic effect when used in combination with trametinib. Such heterogeneity of data can be a consequence of the different power by which each molecule hits its target *in vivo* compared to that one disclosed *in vitro*.

**Table 2:** Mean all grades of each side effect linked to cardiotoxicity of the BRAF inhibitors

Drug	Side effect (mean all grades)				
	Hypertension	Cardiac ischemia / Infarction	Arterial thrombo-embolic events	QT prolongation / ECG changes	CHF and/or symptoms related (e.g. Asthenia, Dyspnea, Peripheral edema)
Sorafenib	~ 22.6 %	~ 2.5 %	~ 1.7 %	~ 8.1 %	~ 14.5 %
Vemurafenib	6 %	------	------	~ 8.5 %	~ 8.8 %
Regorafenib	~ 37.5 %	~ 1.2 %	~ 2 %	No clinically significant effect	6 %
Dabrafenib	------	------	------	------	------

Herein we propose a mechanism through which BRAF inhibitors may determine their cardiotoxic effect. Indeed we hypothesize that BRAF inhibitors blocking this molecule lead to an increase in cAMP activity and through PKA, this event increases hERG channels phosphorylation. These channels normally facilitate K^+^ ions transit, which correlates with myocardial repolarization process. Phosphorylation decreases the function of these channels. Certainly many molecules could intervene in this mechanism. Some data about the association of Raf-1 with cardiovascular adverse events are reported in literature [91, 92].

Although the cardiac effects of BRAF inhibitors in clinical trials are reported in low percentage, attention must be paid on the use of these drugs, because an uncontrolled QT prolongation could expose to a syndrome that would lead to fatal arrhythmias and sudden cardiac death.

Therefore a cooperation between Oncologists and Cardiologists is very important when these drugs are delivered. While Oncologists should pay attention to the potential onset of these effects, Cardiologists should promptly and appropriately treat as necessary the side effects that occur. The rising integrated discipline called Cardio-Oncology should focus on research about BRAF inhibitors, either developing experimental models on cardiotoxicity and planning prospective studies focused on the cardiac effects of these drugs.
